# Correction to: Going beyond consensus genome sequences: An innovative SNP-based methodology reconstructs different Ugandan cassava brown streak virus haplotypes at a nationwide scale in Rwanda

**DOI:** 10.1093/ve/veaf003

**Published:** 2025-01-27

This is a correction to: Chantal Nyirakanani, Lucie Tamisier, Jean Pierre Bizimana, Johan Rollin, Athanase Nduwumuremyi, Vincent de Paul Bigirimana, Ilhem Selmi, Ludivine Lasois, Hervé Vanderschuren, Sébastien Massart, Going beyond consensus genome sequences: An innovative SNP-based methodology reconstructs different Ugandan cassava brown streak virus haplotypes at a nationwide scale in Rwanda, *Virus Evolution*, Volume 9, Issue 2, 2023, vead053, https://doi.org/10.1093/ve/vead053

The originally published version of this manuscript omitted to designate Chantal Nyirakanani and Hervé Vanderschuren as corresponding authors. This error has been corrected.

